# Rare Isolated Pectoralis Minor Tear from a Noncontact Injury: Case Report and Review of the Literature

**DOI:** 10.1155/2019/3605187

**Published:** 2019-08-29

**Authors:** Danica D. Vance, Usama Qayyum, Charles M. Jobin

**Affiliations:** The Center for Shoulder, Elbow and Sports Medicine, Columbia University Medical Center, New York, NY, USA

## Abstract

Isolated pectoralis minor tears are rare orthopedic injuries often in weightlifters or contact sports and should be included in the differential when evaluating athletes with anterior shoulder pain. These injuries are often mistaken for pectoralis major muscle stains and tears. Advanced imaging with MRI helps define the anatomic location and grade of injury. Treatment is usually conservative with full return to activity after a brief period of rest for a few weeks. The purpose of this article is to present a case report and review of the literature on the clinical evaluation, imaging, and treatment of an isolated pectoralis minor tears.

## 1. Introduction

Isolated pectoralis minor tears are rare orthopedic injuries with only a handful of cases reported in the literature. Musculoskeletal shoulder pain localized to the anterior chest and shoulder can be a diagnostic dilemma. The differential diagnosis includes contusion from trauma, costochondritis, muscle strain, pectoralis major tendon tear, and internal derangements of the shoulder. Pectoralis major tears occur from a forced adduction and internal rotation injury often in weightlifters or contact sports [1]. Although rare, isolated pectoralis minor tears may occur from abnormal scapular forces or fatigue of scapular stabilizing musculature and should be included in the differential when evaluating patients with anterior shoulder pain, especially those with tenderness over the coracoid, or lateral chest wall pain near the shoulder [2].

Previous case reports of isolated pectoralis minor tears have described injuries occurring during a contact sport such as American football [2, 3] and ice hockey [4]. In contrast, our case report describes an isolated pectoralis minor tendon tear sustained in a 24-year-old female while she was performing a side plank exercise presumably from loss of endurance and an eccentric loading of her pectoralis minor tendon. These injuries are often mistaken for the more common pectoralis major muscle stains and tears. The purpose of this article is to present the clinical evaluation, imaging, and treatment of an isolated pectoralis minor tear in a young healthy individual sustained from a noncontact injury.

## 2. Case History

A 24-year-old left hand dominant female, smoker for the last five years without a significant medical history, presented one day after an injury at the gym with left shoulder pain. She reports, while preforming a side plank, a maneuver where her body weight is supported by her arm in a side pushup-like position; she felt a pop and snap in her front shoulder and chest area. At the time of injury, the patient had her complete body weight on her fully extended left arm. She had immediate pain without any associated numbness and tingling. The pain did not improve and started to radiate to the neck and down her anterior chest and arm.

Initial physical examination revealed mild asymmetry of the left anterior axillary crease without any gross deformity or ecchymosis. She had tenderness over the left biceps groove and pectoralis major tendon with pain on resisted arm internal rotation. Her brachial plexus and axilla were nontender. Active scapular protraction and retraction were painful. Her coracoid tip was tender to palpation. The remainder of the shoulder exam, including range of motion and neurovascular exam, was unremarkable.

## 3. Imaging

**Figure 1:**
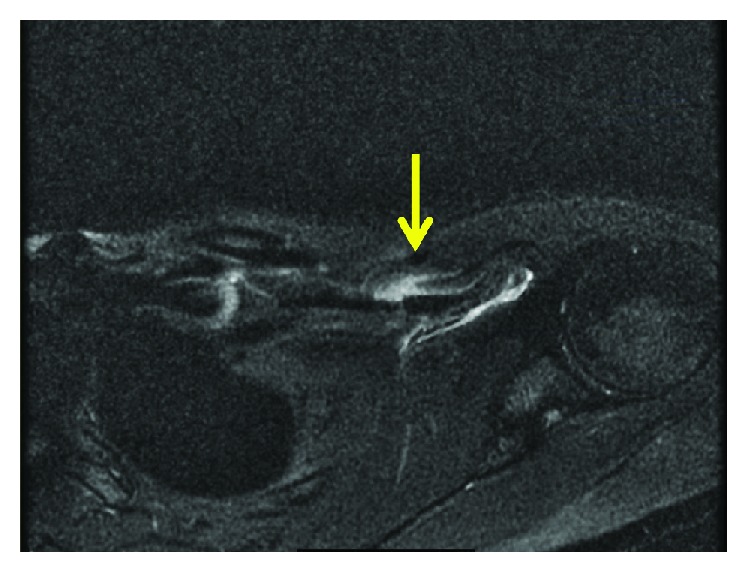
Axial T2-weighted magnetic resonance imaging showing disruption of the pectoralis minor myotendinous junction with surrounding edema and associated intramuscular edema.

Radiographs of her shoulder were unremarkable. Due to the abnormal exam findings and concern for a pectoralis major high-grade muscle strain, a magnetic resonance imaging (MRI) study was ordered. The MRI revealed a grade 3 myotendinous tear of the pectoralis minor with complete disruption of the muscle fibers and visible gaping of the tissue. Additionally, imaging demonstrated surrounding fluid at the myotendinous junction of the pectoralis minor with adjacent muscular edema (Figure 1). The pectoralis major myotendinous complex and rest of the surrounding soft tissue showed no injury.

## 4. Treatment

This injury was managed with conservative treatment including rest, icing, anti-inflammatories, and activity modification. After 2 weeks, the patient recovered to 90 percent without complaints. By 3 weeks, the patient had resumed light workouts with aerobics and dance. At 3-month follow-up, the patient reported no residual disability with full return to all sporting activities. At six-month follow-up, the clinical exam demonstrated an excellent outcome with a physical exam symmetric to the uninjured arm and shoulder. Patient subjective pain score and shoulder assessment scores were as follows: ASES = 100, SST = 12, and constant score = 95. Patient SF-36 scores following nonoperative treatment were high for all categories (physical function = 100%, physical health = 100%, emotional role = 100%, energy/fatigue = 75%, emotional well‐being = 88%, social functioning = 100%, pain = 90%, general health 86%, and health change 75%). The patient had a full painless range of motion of 175° of forward elevation, 65° of external rotation (ER), T5 internal rotation (IR), and strength that was 5/5 and symmetric to the contralateral shoulder. She had no scapular winging or weakness in scapular protraction and retraction.

## 5. Discussion

Pectoralis minor injuries are rare and may be caused by both contact and noncontact traumatic events. The mechanism of injury can be characterized by an eccentric load in a fatigued pectoralis minor muscle. Conservative treatment has been successful in the few case reports of this unique injury.

The pectoralis minor tendon originates from the outer surface of the third through the fifth ribs. Its fibers pass superiorly and laterally and converges to a flat tendon that inserts on the medial superior border of the coracoid process, adjacent to the conjoint tendon [2]. The primary role of the pectoralis minor tendon is to stabilize the scapula by drawing it anteriorly and inferiorly over the thoracic wall [5].

The exact mechanism of pectoralis minor tendon rupture is unknown. Various factors such as excessive strain, abnormal loading, and eccentric forces on the muscle play crucial roles. Injury to the pectoralis minor can occur with direct force to the shoulder or forced ER of the arm [3]. Skeletal muscle is a continuous adapting tissue that responds to stress by changing its physiological function and mass. However, acute stresses that exceed the adaptability leads to injury [6].

Injury to the pectoralis minor tendon can occur through contact injuries in athletes either by direct blows to the anterior shoulder or forced ER of the arm while abducted. The myotendinous junction is the most common sight of injury amongst the current experimental models demonstrating muscle strains [7]. To our knowledge, our case report is the first to describe an injury to the pectoralis minor without contact, as the injury in this case was sustained while the patient was preforming a side plank at the gym. The mechanism of injury was likely due to fatigue of the anterior scapular stabilizers during the eccentric loading of the pectoralis muscles, causing the scapular to be eccentrically forced into retraction.

Physical examination is critical as soft tissue injuries of musculoskeletal origin are elicited by palpating on active trigger points and demonstrating pain or weakness with resisted muscle function. Pec minor injuries present as anterior shoulder pain and tenderness to palpation over the coracoid [3]. Shoulder extension and ER also generate pain [4]. Evaluation of the shoulder at 90° and 150° of horizontal abduction generates tension across the pectoralis minor most effectively [8].

Advanced imaging is recommended if a significant injury is suspected to the pectoralis complex, but no obvious deformity is discovered. A grade 1 strain on imaging is defined as having edema without laxity in any fibers or gapping within the myotendinous complex. Pectoralis minor grade 1 strains have been confused in the past with symptoms of angina [9]. Grade 2 stains demonstrate laxity in fibers indicating significant strain but without tissue gapping. Grade 3 myotendinous tears are defined as a complete disruption of muscle fibers with a visible tissue defect. Other reports of pectoralis minor tears in the literature have revealed tears in the tendon with edema in the muscle mass [3], complete absence of pec minor tendon attachment over the coracoid, and high intensity signal across the entire muscle [4].

Sports medicine literature demonstrates the use of slings, pendulum exercises, and physical therapy for postinjury rehabilitation. A detailed physical clinical exam to exclude any residual injury or disability is performed before athletes start dynamic sport-specific exercises [4]. Conservative treatment has been successful in nearly every patient reported with pectoralis minor injury. Reports of recalcitrant pectoralis tendinopathy have been successfully treated with corticosteroid injections [10]. The patient in this report made a full recovery with a complete normal shoulder with conservative management that included rest, ice, NSAIDs, and activity modification for 2 to 4 weeks.

Pectoralis minor myotendinous injuries are uncommon. When they do occur, the patients are often healthy active individuals, including professional athletes and those who play contact sports. However, pectoralis minor injuries should always be considered when a patient presents with atypical anterior musculoskeletal shoulder and chest wall pain as noncontact mechanisms of injury do occur. Pain and tenderness near the coracoid along with physical exam findings of painful resisted scapular protraction should alert the orthopedist to a possible pectoralis minor injury. Advanced imaging with MRI helps define the anatomic location and grade of injury. Treatment is usually conservative with full return to activity after a brief period of rest for a few weeks.
